# Extension of Duplexed Single-Ended Distributed Temperature Sensing Calibration Algorithms and Their Application in Geothermal Systems

**DOI:** 10.3390/s22093319

**Published:** 2022-04-26

**Authors:** Matías Lillo, Francisco Suárez, Mark B. Hausner, Gonzalo Yáñez, Eugenio A. Veloso

**Affiliations:** 1 Departamento de Ingeniería Hidráulica y Ambiental, Pontificia Universidad Católica de Chile, Santiago 7820436, Chile; malillo@uc.cl; 2 Centro de Desarrollo Urbano Sustentable (CEDEUS), Pontificia Universidad Católica de Chile, Santiago 7820436, Chile; 3 Centro de Excelencia en Geotermia Andina (CEGA), Pontificia Universidad Católica de Chile, Santiago 7820436, Chile; gyanez@ing.puc.cl (G.Y.); eaveloso@gmail.com (E.A.V.); 4 Desert Research Institute, Reno, NV 89512, USA; mark.hausner@dri.edu; 5 Departamento de Ingeniería Estructural y Geotécnica, Pontificia Universidad Católica de Chile, Santiago 7820436, Chile; 6 School of Ocean Sciences, Pontificia Universidad Católica de Valparaíso, Valparaíso 2340025, Chile

**Keywords:** DTS, geothermal exploration, calibration algorithms, duplexed single-ended, borehole temperature measurements

## Abstract

Fiber-optic distributed temperature sensing (DTS) has been widely used since the end of the 20th century, with various industrial, Earth sciences, and research applications. To obtain precise thermal measurements, it is important to extend the currently available DTS calibration methods, considering that environmental and deployment factors can strongly impact these measurements. In this work, a laboratory experiment was performed to assess a currently available duplexed single-ended DTS calibration algorithm and to extend it in case no temperature information is available at the end of the cables, which is extremely important in geothermal applications. The extended calibration algorithms were tested in different boreholes located in the Atacama Desert and in the Central Andes Mountains to estimate the geothermal gradient in these regions. The best algorithm found achieved a root mean square error of 0.31 ± 0.07 °C at the far end of a ~1.1-km cable, which is much smaller than that obtained using the manufacturer algorithm (2.17 ± 0.35 °C). Moreover, temperature differences between single- and double-ended measurements were less than 0.3 °C at the far end of the cable, which results in differences of ~0.5 °C km^−1^ when determining the geothermal gradient. This improvement in the geothermal gradient is relevant, as it can reduce the drilling depth by at least 700 m in the study area. Future work should investigate new extensions of the algorithms for other DTS configurations and determining the flow rate of the Central Andes Mountains artesian well using the geothermal profile provided by the DTS measurements and the available data of the borehole

## 1. Introduction

The determination of the geothermal gradient is relevant for many applications in a wide range of disciplines [1,2]. In renewable and sustainable energy sources, underground temperatures allow for the determination of local and regional geothermal potential [3], and the source temperature defines low- and high-enthalpy geothermal reservoirs [2,4]. In Earth sciences, geothermal data are required to understand the dynamics of tectonic plates in ridge collision zones [5], to determine the effects of temperature in metamorphic formations [6], and for detecting the reinitiation of volcanic activity [7], among other tasks [8,9,10]. Obviously, actual temperature observations are also needed to develop detailed thermal models [11,12]. Moreover, borehole temperature profiles have been used to determine climate variations in different locations around the globe [13,14,15].

The bottom hole temperature (BHT) method is the most common technique to record borehole temperatures and to use them to estimate the geothermal gradient [3,16,17]. This technique is primarily used in the oil extraction industry, where drilling is common and abundant data are available [18]. Borehole temperatures can also be used to predict static formation temperatures, thermophysical properties, and production parameters in oil reservoirs [19], as well as to determine heat flow maps [20]. However, measurements taken by the BHT method often have poor accuracy, and corrections must be performed to improve these observations [21,22]. For instance, Goutorbe et al. [20] show that most of the correction models lead to a reliable estimation of temperature within ±10 °C. In thermal methods that are used to estimate geothermal gradients, direct temperature–depth relationships are determined and then correlated with properties of the geothermal system, since it is fairly simple to measure near-surface temperatures using airborne or satellite-based measurements [23,24]. However, as a near-surface method, they are limited to shallow depths [25]. For instance, according to Lv et al. [26], the penetration depth determined using satellites could be of ~0.3 m, depending on soil properties and moisture. With their spatial and temporal coverage, Raman spectra fiber-optic distributed temperature sensing (DTS) methods offer significant advantages in the environment over traditional measurements systems [27,28]. DTS has been widely used as an in situ logging technique in oil and gas wells, being the only system that offers a data profile that can be used to identify flow patterns and changes in fluid properties, as well as to monitor the overall integrity of the borehole without intervention [29]. Since the 1990s, this technology has had various uses in geosciences [30,31], environmental sciences [32,33,34,35], ecology [36], glaciology [37], hydrology [38,39,40], hydrogeology [41,42,43], engineering [44,45], and industrial applications [29].

Although DTS systems have been successfully used in many environments, achieving high-resolution data is not trivial, as their precision and accuracy depend on the prescribed spatial and temporal sampling intervals [38], deployment and configuration (e.g., single- or double-ended configurations), and calibration methodologies [46,47,48]. Many practical issues can degrade the data quality, among which are the failure of connectors, splices, sharp bends and strains in the cable, excessive heat, and mechanical damage [29], which can be overcome by developing calibration algorithms [46,49]. Nonetheless, only few investigations have developed these algorithms. Hausner et al. [49] determined efficient ways to use the DTS Raman raw data to improve the precision and accuracy of these systems in duplexed single-ended configurations. Hausner and Kobs [46] developed an approach to identify and correct step losses in single-ended configurations, whereas van de Giesen et al. [47] developed a calibration algorithm for double-ended configurations using information from both ends of the optical fiber (see below for details about the different DTS configurations). Even when non-uniform differential attenuation can be addressed using double-ended configurations, in some situations, only a single-ended configuration can be achieved. For instance, we performed a field campaign to determine the unexplored geothermal gradient in the Central Andes of Chile, which is one of the countries with the largest unexploited geothermal potential in the world [50,51]. Knowing the geothermal gradient in this region is relevant, as it can be used for thermomechanical geological models; it serves as a baseline for geothermal exploitation; and on a broader scale, it provides an extrapolation constraint for crustal/lithospheric rheology. Given logistical constraints during the field campaign, data were collected in a ~1.1 km long borehole using single-ended data with no independent thermal measurement at the bottom of the borehole. This issue did not allow for obtaining an accurate temperature at the end of the borehole, as different calibration algorithms cannot be fully constrained [49], resulting in an uncertainty of more than 4 °C at the far end of the fiber-optic cable. This uncertainty may have important implications for data interpretation. The calibration algorithms developed so far for single-ended configurations assume a uniform differential along the fiber and consequently, distributes the errors along the entire fiber [46,49]. Therefore, extending these algorithms to consider different differential attenuation rates and raising awareness of their existence is important for the scientific community that uses fiber optic DTS systems.

Therefore, the objective of this work is to assess and expand current duplexed single-ended calibration algorithms to reduce the uncertainty of the bottom borehole temperature. We performed laboratory experiments to improve the accuracy and precision of single-ended DTS data by extending the current calibration algorithms, and then we applied these new algorithms to the geothermal data collected in different boreholes located in north and central Chile. The application of the extended algorithms improved the accuracy of the geothermal measurements by one order of magnitude, which reduced the uncertainty in drilling depth by 50% when using DTS methods calibrated with the best algorithm that we found (compared to the BHT approach).

## 2. Raman Spectra DTS Theory

Raman spectra DTS technologies use optical fibers as distributed sensors than can be deployed for more than 40 km with a temperature resolution of less than 1 °C [52,53], or even shorter distances with a correspondingly finer temperature [52]. Current DTS technologies allow a spatial sampling resolution on the order of centimeters [54]. The instruments used in this research have a minimum spatial integration of 0.25 and 1 m for fibers up to 1000 m long, and a temporal integration as short as 2 and 10 s [38], although all these specifications cannot be obtained at the same time [28]. To estimate the thermal profile along a fiber-optic cable, a DTS instrument sends a laser pulse into the fiber and measures the backscattered Stokes and anti-Stokes intensities, which have information about the temperature at the location where the backscatter occurred. Then, optical time reflectometry is used to determine the position where backscatter occurred [49]. Therefore, the temperature *T*(K) at position *z* (m) in the cable is expressed as [28,49]:

(1) $$T\left(z\right)=\frac{\gamma }{C+R\left(z\right)-\Delta \alpha \;z}$$

where *γ* (K) represents the shift in energy between a photon at the wavelength of the incident laser and the scattered Raman photon, *C* (-) is a calibration parameter that encompasses properties of the incident laser and the DTS instrument itself, $R\left(z\right)=ln\left(I_{S}\left(z\right)/I_{aS}\left(z\right)\right)$ is the natural logarithm of the ratio between the Sokes and anti-Stokes intensities, *I_S_*(*z*) and *I_aS_*(*z*), respectively, and ∆*α* (m^−1^) is the differential attenuation rate between the backscattered Stokes and anti-Stokes intensities. Since a detailed description of the DTS theory can be found elsewhere [49,55], below we describe the different DTS configurations, with emphasis on duplexed single-ended configurations and their current calibration algorithms, as well as describe how these algorithms are extended and applied to determine the geothermal profile in different Chilean Andean settings.

### 2.1. DTS Configurations and Current Calibration Algorithms

**Figure 1:**
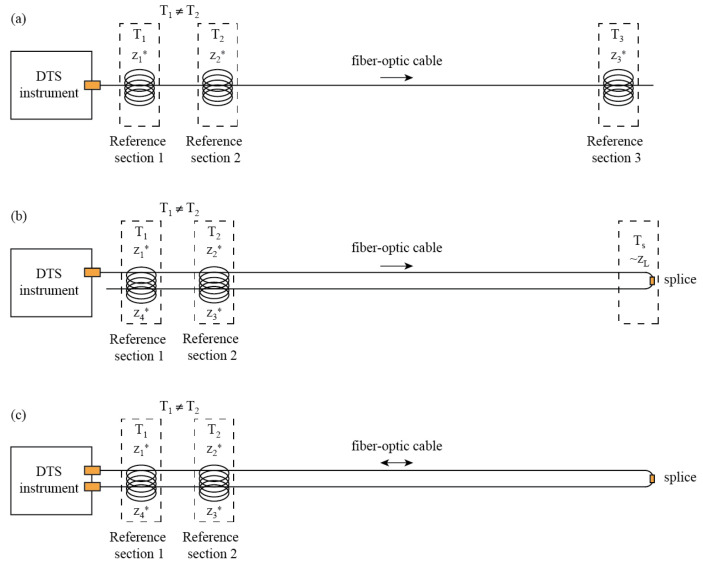
Typical DTS configurations. (**a**) Simple single-ended configuration. (**b**) Duplexed single-ended configuration. (**c**) Duplexed double-ended configuration. Modified from Hausner et al. [49].

There are three typical DTS configurations: simple single-ended, duplexed single-ended, and duplexed double-ended configurations [49] (Figure 1). The simple single-ended configuration uses only one connection to the DTS instrument, and the temperature is measured along the fiber from the DTS onwards. The duplexed single-ended configuration also uses only one connection to the DTS instrument, but there are two co-located fibers that measure temperature with two observations at every section along the cable. This configuration is made by deploying the cable in such a way that the fiber goes out from the DTS instrument and then comes back towards the instrument following the same path, without connecting the end of the cable into the instrument, or by using a cable with two co-located fibers spliced at the far end. The duplexed double-ended configuration is similar to the duplexed single-ended configuration, but with both of the co-located fibers connected to the DTS instrument, and the instrument measuring from alternating ends of the fiber. Thus, temperature observations are performed from both directions within the fiber.

Many DTS instruments have proprietary algorithms provided by the manufacturers that use some form of Equation (1) to calibrate the thermal profile along the cable (also known as the temperature trace). As suggested by Hausner et al. [49], three reference sections of known temperature are needed to properly calibrate single-ended measurements using Equation (1), as long as the differential attenuation is uniform over the section of the fiber where the temperature is being measured. Any additional reference sections will provide independent zones, where the calibration algorithms can be assessed using calibration metrics, as described below. Moreover, calibration algorithms should also consider the existence of step losses. Step losses consist of a signal reduction that is reflected in a sharp drop in the Raman spectra recorded by the DTS instrument [32,46], thus the ratio between the Stokes and anti-Stokes intensities changes with respect to a situation without fiber damage. Hausner and Kobs [46] present a method based on Equation (1) to correct the impact of step losses on estimated temperatures, although it does not consider the effect of having fiber sections with different differential attenuation, which is common when two fibers are fused to create a duplexed cable.

The general procedure to calibrate DTS temperature traces is to find the values of *γ*, *C*, and ∆*α* using the data collected by the DTS combined with independent temperature measurements at known positions along the fiber, i.e., in the reference sections. For instance, Hausner et al. [49] obtain the temperature trace using the explicit calibration method (hereafter referred to as Algorithm 1) by solving a system of three equations written in matrix form:

(2) $$\bar{A}=\left[\begin{array}{ccc} 1 & -T\left(z_{1}\right) & T\left(z_{1}\right)\;z_{1}\\ 1 & -T\left(z_{2}\right) & T\left(z_{2}\right)\;z_{2}\\ 1 & -T\left(z_{3}\right) & T\left(z_{3}\right)\;z_{3} \end{array}\right]\;\vec{x}=\left[\begin{array}{c} \gamma \\ C\\ \Delta \alpha \end{array}\right]\;\vec{b}=\left[\begin{array}{c} T\left(z_{1}\right)\;R\left(z_{1}\right)\\ T\left(z_{2}\right)\;R\left(z_{2}\right)\\ T\left(z_{3}\right)\;R\left(z_{3}\right) \end{array}\right]$$

(3) $$\bar{A}\vec{x}=\vec{b}$$

where the subindices correspond to the reference sections with independent temperature measurements (see Figure 1). To obtain the best calibration, it is recommended that each reference section should have at least ten observations performed using the DTS system [32,49,55]. When *n* DTS observations are within a reference section, the mean distance of the reference section, $z^{*}=1/n\overset{n}{\underset{i=1}{\sum }}z_{i}$, and the mean natural logarithm of the ratio between *I_S_*(*z*) and *I_aS_*(*z*), i.e., $R{\left(z\right)}^{*}=1/n\overset{n}{\underset{i=1}{\sum }}R\left(z_{i}\right)$, should be used to achieve the best results [49].

Step losses or singularities occur frequently in field deployments due to impingements on the fiber, sharp bends, or splices [46], and it is important to identify and remove them before performing the calibration using Equations (2) and (3). Hausner and Kobs [46] propose that step losses can be identified by visual inspection, or by analyzing changes in the variance of *I_S_*(*z*) and *I_aS_*(*z*). Because the anti-Stokes signal is more sensitive to temperature changes compared to the Stokes signal, step losses that occur in sections with a constant temperature may result in abrupt reductions of similar magnitude in both the Stokes and anti-Stokes signal. To remove the singularities, Hausner and Kobs [46] propose to calculate the difference of R(z) at both sides of the singularity using one or more points. For instance, if the singularity is located at $z_{L}$ (e.g., due to a splice, such as that shown in Figure 1b), then $\Delta R=R{\left(z_{L-1}\right)}^{*}-R{\left(z_{L+1}\right)}^{*}$ for a section with one or more points, in which the subindices L−1 and L+1 refer to the fiber sections before and after the step loss, respectively. To correct the temperature trace, a new value of R(z) must be calculated after the step loss:

(4) $$R\left(z>z_{L}\right)=R\left(z\right)+\Delta R,$$

### 2.2. Extended Calibration Algorithms

Given that the previous algorithms assume a uniform differential attenuation rate throughout the fiber-optic cable for a single-ended configuration, and considering that the double-ended configuration is not always feasible, we extended the current algorithms to consider two different differential attenuations in a duplexed single-ended configuration. These extensions are thought to work when the temperature at the end of the cable is unknown, and all of them also correct for step losses using the method proposed by Hausner and Kobs [46] before applying the algorithms.

#### 2.2.1. Calibration by Sections (Algorithm 2)

This algorithm is a slight modification of Algorithm 1 (Hausner et al. [49], explicit method) aimed to improve the calibration of $T\left(z>z_{L}\right)$. It consists of calibrating $T\left(z<z_{L}\right)$ using Algorithm 1 and reference sections from both sides of the cable, e.g., $T\left(z_{1}^{*}\right)$, $T\left(z_{2}^{*}\right)$ and $T\left(z_{3}^{*}\right)$ (see Figure 1b). From this calibration, the temperature just before $z_{L}$, $T\left(z_{L}-\Delta z\right)$, is used as a reference temperature in the next location after the step loss, i.e., $T\left(z_{L}-\Delta z\right)=T\left(z_{L}+\Delta z\right)$, where Δz is a distance at which the effect of the splice is not seen. Then, the calibration of $T\left(z>z_{L}\right)$ uses Algorithm 1 with the following reference locations: $T\left(z_{L}+\Delta z\right)$, $T\left(z_{3}^{*}\right)$, and $T\left(z_{4}^{*}\right)$. Note that this algorithm assumes that the differential attenuation of both sides of the cable is identical.

#### 2.2.2. Explicit Calibration Using Two Differential Attenuations (Algorithm 3)

When a duplexed single-ended configuration is made by fusing two different fibers, it is likely that their differential attenuation will differ. Therefore, a logical and simple extension of Algorithm 1 is to use $\Delta \alpha_{1}$ and $\Delta \alpha_{2}$ as the differential attenuations for the fiber before and after the step loss, respectively. In the same way as in Algorithm 1, a set of four equations can be written in matrix form, i.e., $\bar{A}\vec{x}=\vec{b}$, to find the calibration parameters *γ*, *C*, $\Delta \alpha_{1}$, and $\Delta \alpha_{2}$:

(5) $$\overset{ˉ}{A}=\left[\begin{array}{cccc} 1 & -T\left(z_{1}\right) & T\left(z_{1}\right)z_{1} & 0\\ 1 & -T\left(z_{2}\right) & T\left(z_{2}\right)z_{2} & 0\\ 1 & -T\left(z_{3}\right) & T\left(z_{3}\right)z_{3} & 0\\ 1 & -T\left(z_{4}\right) & 0 & T\left(z_{4}\right)z_{4} \end{array}\right]\vec{x}=\left[\begin{array}{c} \gamma \\ C\\ \Delta \alpha_{1}\\ \Delta \alpha_{2} \end{array}\right]\vec{b}=\left[\begin{array}{c} T\left(z_{1}\right)R\left(z_{1}\right)\\ T\left(z_{2}\right)R\left(z_{2}\right)\\ T\left(z_{3}\right)R\left(z_{3}\right)\\ T\left(z_{4}\right)R\left(z_{4}\right) \end{array}\right]$$

(6) $$T\left(z<z_{L}\right)=\frac{\gamma }{C+R\left(z\right)-\Delta \alpha_{1}\;z}\;,$$

(7) $$T\left(z>z_{L}\right)=\frac{\gamma }{C+R\left(z\right)-\Delta \alpha_{2}\;z}\;,$$

Note that now after solving Equation (3) with $\bar{A}$ and $\vec{b}$ obtained from Equation (5), temperatures along the fiber are estimated using Equations (6) and (7).

#### 2.2.3. Explicit Calibration Using Two Differential Attenuations and Two γ (Algorithm 4)

This algorithm is an extension of Algorithm 3, in which γ is used as a calibration parameter that can be different before and after the step loss, i.e., now we have $\gamma_{1}$ and $\gamma_{2}$ for each side of the cable. Hence, a set of five equations can be written in matrix form to find the calibration parameters $\gamma_{1}$, $\gamma_{2}$, *C*, $\gamma \alpha_{1}$, and $\Delta \alpha_{2}$:

(8) $$\overset{ˉ}{A}=\left[\begin{array}{ccccc} 1 & 0 & -T\left(z_{1}\right) & T\left(z_{1}\right)z_{1} & 0\\ 1 & 0 & -T\left(z_{2}\right) & T\left(z_{2}\right)z_{2} & 0\\ 1 & 0 & -T\left(z_{3}\right) & T\left(z_{3}\right)z_{3} & 0\\ 0 & 1 & -T\left(z_{4}\right) & 0 & T\left(z_{4}\right)z_{4}\\ 0 & 1 & -T\left(z_{5}\right) & 0 & T\left(z_{5}\right)z_{5} \end{array}\right]\vec{x}=\left[\begin{array}{c} \gamma_{1}\\ \gamma_{2}\\ C\\ \Delta \alpha_{1}\\ \Delta \alpha_{2} \end{array}\right]\vec{b}=\left[\begin{array}{c} T\left(z_{1}\right)R\left(z_{1}\right)\\ T\left(z_{2}\right)R\left(z_{2}\right)\\ T\left(z_{3}\right)R\left(z_{3}\right)\\ T\left(z_{4}\right)R\left(z_{4}\right)\\ T\left(z_{5}\right)R\left(z_{5}\right) \end{array}\right]$$

(9) $$T\left(z<z_{L}\right)=\frac{\gamma_{1}}{C+R\left(z\right)-\Delta \alpha_{1}\;z}$$

(10) $$T\left(z>z_{L}\right)=\frac{\gamma_{2}}{C+R\left(z\right)-\Delta \alpha_{2}\;z}$$

After solving Equation (3) with $\bar{A}$ and $\vec{b}$ obtained from (8), the temperatures throughout the fiber are estimated with Equations (9) and (10).

#### 2.2.4. Explicit Calibration Using Two Differential Attenuations and Two C (Algorithm 5)

This algorithm is also an extension of Algorithm 3, but in which *C* is used as a calibration parameter that can be different before and after the step loss, i.e., now we have $C_{1}$ and $C_{2}$ for each side of the cable. A set of 5 equations can be written in matrix form to find the calibration parameters *γ*, $C_{1}$, $C_{2}$, $\Delta \alpha_{1}$, and $\Delta \alpha_{2}$.

(11) $$\overset{ˉ}{A}=\left[\begin{array}{ccccc} 1 & -T\left(z_{1}\right) & 0 & T\left(z_{1}\right)z_{1} & 0\\ 1 & -T\left(z_{2}\right) & 0 & T\left(z_{2}\right)z_{2} & 0\\ 1 & -T\left(z_{3}\right) & 0 & T\left(z_{3}\right)z_{3} & 0\\ 1 & 0 & -T\left(z_{4}\right) & 0 & T\left(z_{4}\right)z_{4}\\ 1 & 0 & -T\left(z_{5}\right) & 0 & T\left(z_{5}\right)z_{5} \end{array}\right]\vec{x}=\left[\begin{array}{c} \gamma \\ C_{1}\\ C_{2}\\ \Delta \alpha_{1}\\ \Delta \alpha_{2} \end{array}\right]\vec{b}=\left[\begin{array}{c} T\left(z_{1}\right)R\left(z_{1}\right)\\ T\left(z_{2}\right)R\left(z_{2}\right)\\ T\left(z_{3}\right)R\left(z_{3}\right)\\ T\left(z_{4}\right)R\left(z_{4}\right)\\ T\left(z_{5}\right)R\left(z_{5}\right) \end{array}\right]$$

(12) $$T\left(z<z_{L}\right)=\frac{\gamma }{C_{1}+R\left(z\right)-\Delta \alpha_{1}\;z}$$

(13) $$T\left(z>z_{L}\right)=\frac{\gamma }{C_{2}+R\left(z\right)-\Delta \alpha_{2}\;z}$$

After solving Equation (3) with $\bar{A}$ and $\vec{b}$ obtained from (11), the temperatures along the fiber are estimated with Equations (12) and (13).

## 3. Materials and Methods

To assess the previous calibration algorithms, we performed an experiment in the laboratory, and then we tested the algorithm that performed better with DTS data obtained in boreholes located in northern and central Chile. Below, we describe the experimental setup in the laboratory and in the different boreholes, as well as the calibration and validation metrics used to assess the calibration algorithms.

### 3.1. Laboratory Experiment

An experiment was carried out in the Open Channel Laboratory of the Department of Hydraulic and Environmental Engineering of the Pontificia Universidad Católica de Chile. The aim of this experiment was to replicate the deployment of a fiber-optic cable in a borehole using a duplexed single-ended configuration. Moreover, the laboratory experiment used the DTS instrument and fiber-optic cable that were used to observe the borehole in the Chilean Central Andes described below.

**Figure 2:**
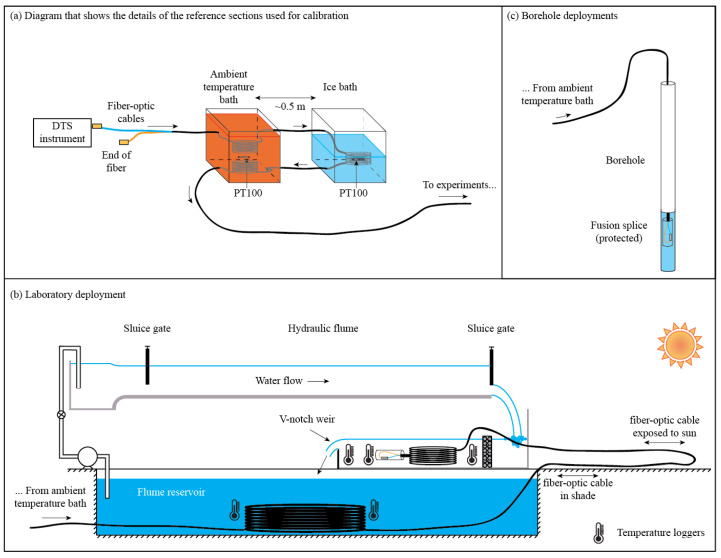
(**a**) DTS instrument and calibration/validation sections of the deployments carried out in this work. (**b**) Laboratory deployment. (**c**) Field deployment in boreholes (northern and central Chile experiments).

Data were collected using a Sensornet Oryx DTS instrument (Sensornet, Hertfordshire, UK), which has two reference thermometers constructed from 100 Ω platinum PT100 sensors. These thermometers have an accuracy of 0.1 °C and a precision of 0.02 °C. The PT100 sensors were installed in two calibration baths to obtain reference sections with an independent temperature measurement, which is required for calibration (see Figure 2a,b). We used a 6-mm outer diameter armored FO PBT patchcord 50/125 duplexed cable (Kaiphone Technology Co., LTD., Taipei, Taiwan) of approximately 1.1 km. In this deployment, ~52 m of the duplexed cable passed through a recirculated water bath at ambient temperature, ~57 m of cable passed through an ice-bath that was at 0 °C, and then ~50 m of cable passed again through the ambient-temperature bath. Being a duplexed configuration, this deployment allows for up to six reference sections. Then, ~58 m of cable were lowered into one of the water reservoirs of the flumes; subsequently, the cable went out of the laboratory so that ~117 m of cable were exposed to sun. Finally, the last ~217 m of cable were immersed in the water upstream of a weir in order to create a final section of uniform temperature due to the turbulent mixing of the water. At the end of the cable, two fibers were fused and protected, creating the duplexed single-ended configuration. Therefore, the second half of the duplexed trace must be the mirror image of the first half, but with a step loss in the middle. We installed 11 HOBO Water Temperature Pro v2 Data Loggers (Onset, Bourne, MA, USA), with an accuracy of 0.2 °C and a resolution of 0.02 °C, at different locations of the fiber to have independent temperature measurements throughout the cable: three were placed in each of the two calibration baths, two in the flume reservoir, and another three upstream of the weir (see Figure 2b). Data were collected for ~6 h using the single-ended mode, with a 1 m sampling interval and 2 min integration time (Table 1).

### 3.2. Field Evaluation

#### 3.2.1. Boreholes in Northern Chile: Revisiting Geothermal Gradients Using Single- and Double-Ended Data

We carried out measurements with an Ultima-XT DTS instrument (Silixa, Hertfordshire, UK) in three different boreholes located in the Atacama Desert (Inca de Oro, Copiapó, and Punto Diaz) using the proposed algorithms to obtain the associated geothermal gradient. This dataset was collected by Pickler et al. [14] using a duplexed double-ended configuration (see Figure 1c), whose fiber-optic cable passed through boreholes with depths ranging between 328 and 572 m. Therefore, the results of the proposed calibration algorithms were compared to the double-ended measurements. Table 1 presents information related to the field site and the instrument configuration of each borehole. In these deployments, the cable was the same as that used in the laboratory experiment, with three calibration baths at the beginning of the cable, which are also seen at its end due to the duplexed nature of the cable (see Figure 2a,c). More details about the location of the boreholes and of the collection methodology is described by Pickler et al. [14].

#### 3.2.2. Borehole in the Chilean Central Andes: Geothermal Gradient

Geothermal gradient measurements were performed in a cased borehole in the División Andina (DAND) de Codelco mine, which is located more than 2700 m ASL in the Central Andes of Chile. The aim of these measurements was to observe the geothermal gradient in an unexplored region of the country, within the flat slab segment of the Nazca Plate Subduction, where no active volcanoes are present [56]. Nonetheless, given difficulties that occurred when accessing the mine, which included a damaged connector and a short time available to perform the measurements, these measurements initially had an uncertainty of ~4 °C at the far end of the fiber-optic cable, which is significant for geothermal gradient estimation.

This deployment also had the same DTS instrument, cable, and duplexed single-ended configuration as the laboratory experiment (see Figure 2a,c). However, due to the difficulties described above, only duplexed single-ended measurements were performed, with independent temperature measurements in the calibration baths. The DTS temperatures were collected with a 1 m sampling interval, a 1 min integration time, and for ~15 min. For the analysis, the data were integrated over 2 min to be consistent with the laboratory data, as well as with the data of the boreholes located in northern Chile (Table 1).

### 3.3. Calibration and Validation Metrics

After processing the DTS data, it is important to estimate both the accuracy and precision of the calibrated data. As suggested by Hausner et al. [49], mean bias *MB* (°C), root mean square error *RMSE* (°C), and duplexing error *E_DUP_* (°C) are used as metrics for the quality of the calibration:

(14) $$MB=\frac{1}{n}\sum_{i=1}^{n}\left(T_{i}-T\right),$$

(15) $$RMSE=\sqrt{\frac{1}{n}\sum_{i=1}^{n}{\left(T_{i}-T\right)}^{2}},$$

(16) $$E_{DUP}=\frac{1}{n}\left|\sum_{j=1}^{n}T_{j,1}-T_{j,2}\right|,$$

where *T_i_* and *T* (°C) are the calibrated and independent temperatures used for calibration and/or validation, respectively, and *T_j_*_,1_ and *T_j_*_,2_ (°C) are the two temperature observations at the same point of the cable when using a duplexed configuration.

## 4. Results and Discussion

### 4.1. Laboratory Experiment and Selection of the Best Algorithm

**Figure 3:**
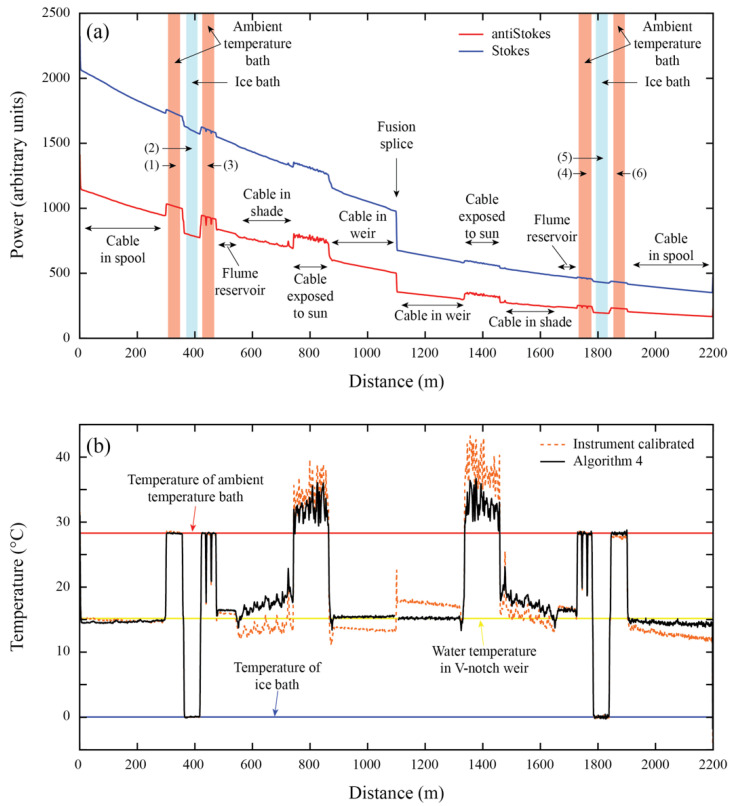
The laboratory deployment. (**a**) Raw Raman spectra data recorded by DTS and the location of the different zones along the fiber-optic cable. (**b**) Calibrated temperature profiles along the fiber-optic cable. The numbers in parentheses in panel (**a**), i.e., (1)–(6), depict a reference section (calibration or validation zone).

A comparison of the metrics between the five algorithms, as well as the manufacturer calibration, is presented in Table 2. The calibration parameters obtained with each algorithm are presented in Table A1 (Appendix A). As expected, the extended calibration algorithms outperform the manufacturer calibration. All the algorithms have a good performance in terms of *RMSE* in the calibration baths, but their accuracy decreases with distance. Temperature differences of ~1.5 °C are observed in the validation bath when using the manufacturer calibration (Figure 3). These differences are reduced to ~0.25 °C when using the extended calibration algorithms.

Algorithm 4 is the one that results in the least biased temperature and smallest *RMSE* (see validation metrics), even when Algorithms 1 and 2 display better metrics in the calibration sections (see calibration metrics). Table 2 also presents the *RMSE* at the weir (see Figure 2b and Figure 3), which corresponds to the furthest location from the DTS instrument. At the weir, Algorithm 4 is the one that displays the best validation metrics. Data correction in a duplexed configuration is best checked by examining the duplexing error [49]. In the case of the laboratory calibration, the smallest duplexed error is reached with Algorithm 2, followed by Algorithm 4.

When a second value of *γ* is introduced for the second half of the cable, a great improvement in terms of *RMSE*, *MB,* and duplexed error is obtained in Algorithms 2 and 4 (see validation metrics in Table 2). This improvement is achieved because *γ*, a physical parameter that should be constant, is used as a calibration parameter, in a similar way to the method carried out by Suárez et al. [55] and Hausner et al. [49]. On the contrary, Algorithm 5 presents the worst value of *E_DUP_* because the algorithm overestimates the temperatures at the far end of the second section. Hence, using a different value of *C* for each side of the fiber does not improve results.

### 4.2. Field Evaluation

#### 4.2.1. Boreholes in Northern Chile: Revisiting Geothermal Gradients Using Single- and Double-Ended Data

**Figure 4:**
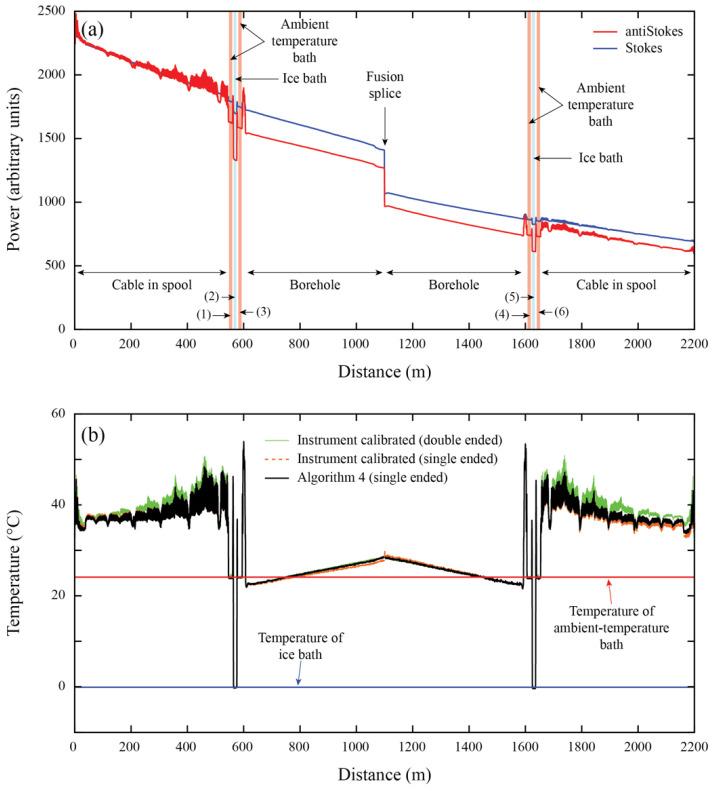
The northern Chile borehole deployments. As an example, borehole DDH2457 is presented: (**a**) Raw Raman spectra data recorded by DTS and the location of the different zones along the fiber-optic cable. (**b**) Calibrated temperature profiles along the fiber-optic cable. The numbers in parentheses shown in panel (**a**), i.e., (1)–(6), depict a reference section (calibration or validation zone).

Table 3 shows a comparison between the quality metrics obtained using Algorithm 4 in the northern Chile dataset, comparing the double and single-ended temperatures of each borehole (see also Figure 4). Table A2 (Appendix A) also presents the calibration parameters obtained in each borehole. For this comparison, the comparative error (*E_COMP_*) is defined as the error between single- and double-ended measurements, using the following expression:

(17) $$E_{COMP}=\frac{1}{n}\left|\sum_{j=1}^{n}T_{i}^{SE}-T_{i}^{DE}\right|,$$

where $T_{i}^{SE}$ and $T_{i}^{DE}$ (°C) are the calibrated temperatures of the single- and double-ended measurements at the same location in the fiber.

**Figure 5:**
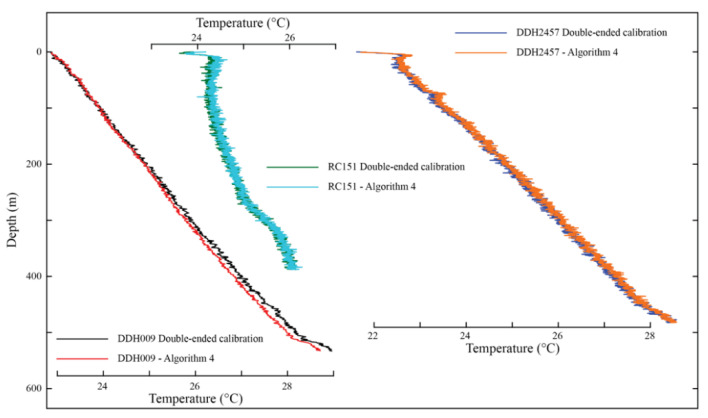
Geothermal profiles measured in the boreholes located in northern Chile (boreholes ID DDH009, RC151, and DDH2457).

In general, the results presented in Table 3 show a good performance of Algorithm 4, compared to the double-ended temperatures in terms of *RMSE*, *MB*, *E_DUP_*, and *E_COMP_*. This good performance is also seen in Figure 5, where the thermal profile of each borehole, obtained with single- and double-ended measurements, is presented. As shown in Table 3, Borehole DDH2457 present a slightly better metrics in the calibration zones for the single-ended dataset, with all the quality metrics being less than 0.1 °C. Borehole RC151 has better quality metrics in the single-ended dataset compared to the double-ended dataset, with a difference of ~0.1 °C in the calibration zones and a similar *RMSE* in the validation zone. Furthermore, the *E_COMP_* of both RC151 and DDH2457 boreholes indicates a good accuracy between single- and double-ended measurements, with an average difference of 0.065 °C and 0.073 °C, respectively. The agreement between single- and double-ended measurements in borehole DDH2457 can also be observed in Figure 4. The single-ended dataset presents a higher *E_DUP_* than the double-ended dataset, a situation that has been improved by removing the unusual values between the environmental changes, as done by Hausner et al. [49]. Finally, borehole ID DDH009 has the worst agreement between single- and double-ended measurements, as reflected by its large *E_COMP_*, although this value is still within acceptable limits.

Table 4 presents the geothermal gradients estimated in these boreholes by Pickler et al. [14] using the double-ended configuration, and those gradients estimated with Algorithm 4 (single-ended measurements). A difference of 0.1 °C km^−1^ is obtained in borehole RC151, showing the best agreement between both datasets in all these boreholes. Boreholes DDH2457 and DDH009 display a difference of 0.5 °C km^−1^ in the geothermal gradient, or approximately 5% of the estimated gradient. In all cases, the geothermal gradient estimated using single-ended measurements (Algorithm 4) underestimates the geothermal gradient determined using double-ended measurements. The differences in the temperatures at the cable’s end obtained with single- and double-ended configurations are less than 0.3 °C, with differences of 0.1 °C, 0.04 °C, and 0.26 °C for the boreholes DDH2457, RC151, and DDH009, respectively. While for practical purposes, such as the determination of geothermal potential, the difference between the geothermal gradients is not large, temperature is one of the main geological variables, and it determines whether shallow temperatures are sufficient for conventional geothermal energy extraction [57]. The optimal temperature depends on the intended usage of the extracted energy. For example, temperature resources less than 150 °C are used for direct heating, whereas temperatures greater than 150 °C are used for electricity generation [58]. Considering a surface temperature of 20 °C and an average geothermal gradient of 10 °C km^−1^ (such as those obtained in the monitored boreholes), a difference of ~0.5 °C km^−1^ in the geothermal gradient estimation results in an uncertainty of the drilling depth of at least of 750 m to reach 150 °C. If a typical (average) geothermal gradient of 25 °C km^−1^ [8] and the same surface temperature of 20 °C are considered, the same difference of ~0.5 °C km^−1^ results in an uncertainty of ~100 m in the drilling depth to reach the same 150 °C. In contrast, considering that the errors in the geothermal gradient calculation using the BHT method could vary in 0.9 °C km^−1^, depending on the method used for the correction of the dataset [16], an uncertainty of ~200 m in the drilling depth for an average geothermal gradient of 25 °C km^−1^, and an uncertainty of ~1500 m for a low geothermal gradient of 10 °C km^−1^, are obtained. Therefore, the developed algorithms allow for the reduction in the uncertainty in the drilling depth of 50% compared with the BHT method.

#### 4.2.2. Borehole in the Chilean Central Andes: Geothermal Gradient

**Figure 6:**
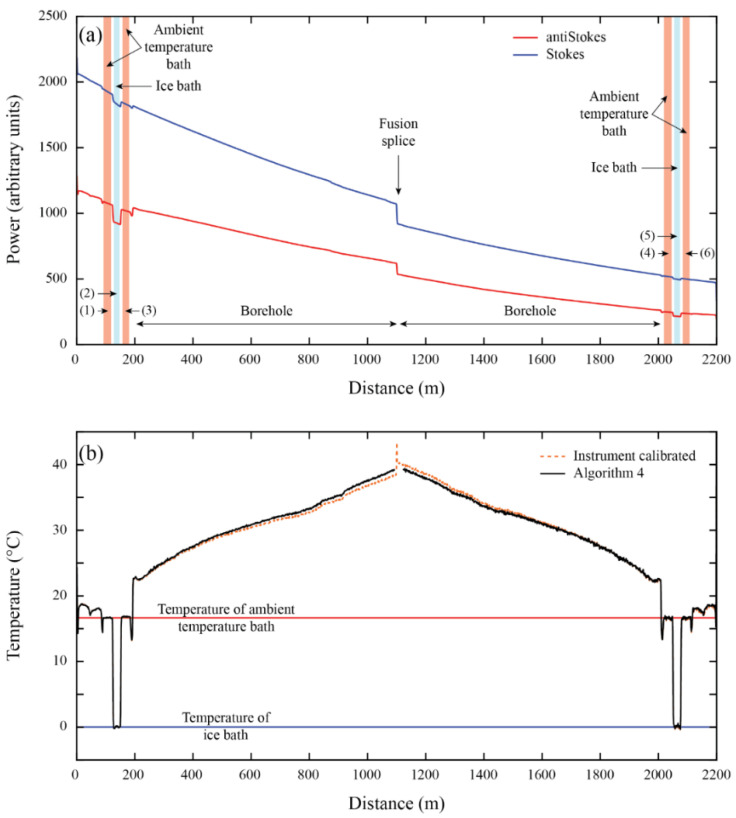
The Central Andes deployment: (**a**) Raw Raman spectra data recorded by DTS and the location of the different zones along the fiber-optic cable. (**b**) Calibrated temperature profiles along the fiber-optic cable. The numbers in parentheses shown in panel (**a**), i.e., (1)–(6), depict a reference section (calibration or validation zone).

**Figure 7:**
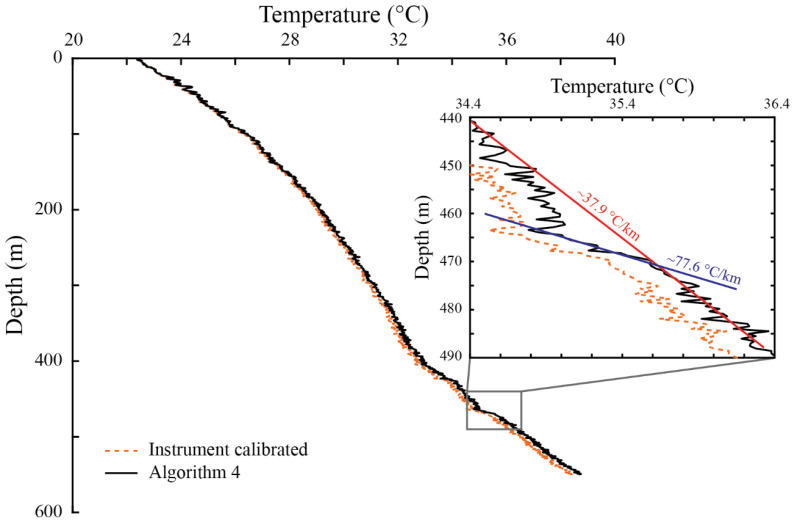
Geothermal profile measured in the DAND borehole located in the Central Andes of Chile.

Table 5 shows the metrics obtained using Algorithm 4 in the Central Chilean Andes dataset (DAND borehole), and Table A2 (Appendix A) presents its calibration parameters. Figure 6 presents the DTS raw data and the thermal profiles obtained with the manufacturer calibration and with Algorithm 4 along the fiber-optic cable, and Figure 7 presents the temperature profile measured in the DAND borehole.

In the validation section, *RMSE* and *MB* are lower than 0.2 °C, whereas the duplexed error is slightly larger (0.22 °C). The *RMSE* in the validation section is smaller than that obtained in the laboratory setup (Table 2) and in the DDH009 borehole (Table 3), but larger than those obtained in the RC151 and DDH2457 boreholes (Table 3). The *MB* is smaller than that obtained in the laboratory deployment (Table 2), but larger than those of the other boreholes (Table 3). Nonetheless, all these errors are much lower than those obtained with the typical calibration algorithms: an uncertainty in the estimated temperature at the cable’s end of 4 °C in the DAND borehole was estimated with the different algorithms developed previously. Considering the deployment made in the laboratory, it is possible to improve the temperature estimation in the far end of the cable using the proposed Algorithm 4. This improvement could not be achieved without performing the independent laboratory experiment, which had a similar deployment configuration to that of the DAND borehole.

In the DAND borehole, after correcting for the dip, we estimated a geothermal gradient of ~37.9 °C km^−1^. This geothermal gradient was determined using the temperature data from the last 170 m of cable, which exhibited a linear trend. The vertical distance of the last 170 m of cable are equivalent to 105 m in the vertical direction, as the borehole was inclined. This geothermal gradient is consistent with the normal (average) geothermal gradient of the Earth’s surface in a normal crust within the first 3 km and away from volcanic sources (the nature of the flat slab segment in which this borehole was located) [8]. Valdenegro et al. [11] reported a borehole gradient of 20 °C km^−1^, but their work assumed the borehole was vertical and did not correct for the drilling dip. However, a larger thermal gradient in the area is in better agreement with the Valdenegro et al. [11] model from this region (above 25 °C km^−1^).

At ~460–480 m depth, cool water flowing through a confined aquifer was detected (see zoom in Figure 7). This water did not enter the borehole, as it was cased, but perturbed the geothermal profile. This cool-water aquifer locally increased the geothermal profile up to ~77.6 °C km^−1^, which cannot be explained by changes in the thermal properties of the rock, as the borehole was drilled in granodiorite, which has a relatively uniform thermal conductivity of ~1.8 W m^−1^ K^−1^ [51]. The water input that entered at the bottom of the cased borehole flows upward, emerging through the upper part of the well, modifying the temperature profile at shallow depths, in which a parabolic shape is observed (Figure 7).

The significance of the improvement in the determination of the temperature at the cable’s end can also be explained using the example presented in the previous section, in which one would like to find the depth where a temperature of 150 °C occurs to define the borehole depth required for electricity generation. In the DAND borehole, the temperature at ~500 m depth is of ~37.9 °C (Figure 7). Assuming an error of ~4 °C in that temperature, and a geothermal gradient of ~37.9 °C km^−1^, the depth where 150 °C is achieved is ~3 km, and the uncertainty in this depth is ~100 m. This uncertainty is reduced to ~10 m when the error in the temperature at the end of the cable is ~0.2 °C. Moreover, if the geothermal gradient in the DAND borehole has an uncertainty of ~0.1 °C km^−1^, errors of ~4 and 0.2 °C in the estimation of the temperature at the cable’s end result in uncertainties of ~120 and ~25 m, respectively, for the depths required to achieve the 150 °C. Therefore, this method provides a reliable tool to obtain the geothermal gradient with confidence, so the implementation of new algorithms to improve DTS temperature estimations are important and must be considered in any field deployment.

### 4.3. Limitations of the Proposed Extended Algorithms

As demonstrated above, the proposed extended DTS calibration algorithms greatly improve the accuracy and precision of thermal measurements along the fiber-optic cable when no temperature information is available at the end of the cables. Nonetheless, these algorithms have limitations that must be considered when they are applied.

The main limitation is related to the assumption of having a uniform differential attenuation at each section of the cables. Even when the proposed algorithms consider that the two fibers co-located in a single cable can have different differential attenuation between each other, they are unable to improve the thermal measurements along fibers that have spatially distributed differential attenuations. For such situations, double-ended configurations are the most appropriate approach to calibrate DTS temperatures, as the fibers are interrogated from both sides and, consequently, differential attenuation can be resolved at every segment of the fiber [47]. This situation cannot be successfully resolved using single-ended data, unless step losses are the sole reason for observing unexpected variations in the raw data. In this case, it is critical to remove the step losses using the approach developed by Hausner and Kobs [46] before applying the methods proposed in this research.

The second limitation is associated to the fact that some of the parameters used in the calibration process do not necessarily fulfill physical considerations; hence, physical parameters are converted into calibration parameters. For instance, most of the algorithms use γ as a calibration parameter instead of the shift in energy between a photon at the wavelength of the incident laser and the scattered Raman photon. Nonetheless, this issue also applies to previously developed methods [49].

Regarding limitations related to the deployment itself, the proposed calibration algorithms require at least four reference baths, with at least two different temperatures. As this research is focused on duplexed single-ended configurations, this is not a significant issue, as these reference sections will be located near the DTS instrument.

## 5. Conclusions

Geothermal exploration requires obtaining accurate measurements at the bottom of boreholes, especially if a certain temperature must be reached in the drilling process to exploit the geothermal resource. An accurate measurement will significantly reduce the existing uncertainty related to the rock temperature at a given depth. Our results show that the uncertainty in drilling can be reduced by 50% when using DTS methods (compared to the BHT approach).

Calibrated single- and double-ended temperature data in the northern Chile boreholes had similar results at the far end of the cable, with differences of up to 0.3 °C. Although the double-ended configuration is preferable, as it does not require a knowledge of the temperature at the end of the cable, the developed algorithms reduced the uncertainty compared to the already existing algorithms in cases when double-ended data are not possible.

Different fiber optic DTS calibration algorithms, including the manufacturer calibration, have a good performance in the first meters of the cable, considering the calibration and validation zones, but an analysis of the last meters of the cable (>1000 m) shows that the temperature difference is as much as 2.5 °C. Considering only existing and proposed algorithms, it is possible to improve the accuracy up to 0.25 °C in the cable’s last meters. This improvement is possible when calibration regions are located in both sections of the fiber. Moreover, a further reduction in the calibration uncertainty can be achieved when an independent temperature measurement is available at the end of the fiber.

Future work should investigate new extensions of the algorithms for other DTS configurations and determining the flow rate of the DAND artesian well using the geothermal profile provided by the DTS measurements and the available data of the borehole.

## Figures and Tables

**Table 1:** Description of the measurements made in the laboratory and field campaigns. The Inca de Oro, Copiapó, and Punta Díaz locations correspond to the DTS datasets collected by Pickler et al. [14]. All the deployments have a sampling interval of 1 m and an integration time of 2 min.

Site	Coordinates	Configuration	Log ID	Observations	Traces
Laboratory		Single-Ended	DIHA	2168	188
Inca de Oro	26°45′10.8″ S 69°53′38.4″ W	Single-Ended	DDH2457	2187	8
		Double-Ended		2187	7
Copiapó	27°22′55″ S 70°13′27″ W	Single-Ended	DDH009	2187	16
		Double-Ended		2187	5
Punta Diaz	28°01′56.3″ S 70°38′44.2″ W	Single-Ended	RC151	2187	5
		Double-Ended		2187	4
División Andina de Codelco	33°4′54″ S 70°15′18″ W	Single-Ended	DAND	2168	7

**Table 2:** Calibration and validation metrics for the laboratory deployment. Above: calibration metrics for the reference temperature baths. Below: calibration metrics for the weir in the far end of the fiber and validation metrics (known independent measures temperatures not used in the calibration, i.e., weir and temperature baths not used in the calibration metrics). The location of the calibration baths, i.e., *z*_1_–*z*_6_, are depicted in Figure 3.

Calibration Algorithm	Calibration Metrics			
	RMSE (°C)μ±σ (range)	MB (°C)μ±σ (range)	Calibration baths	
Manufacturer calibration	0.727±0.648(0.078 to 1.375)	−0.338±0.451(−0.789 to 0.113)	*z*_1_, *z*_2_, *z*_5_	
1	0.098±0.051 (0.047 to 0.150)	−0.004±0.021(−0.016 to 0.025)	*z*_1_, *z*_2_, *z*_5_	
2	0.120±0.058(0.061 to 0.178)	−0.004±0.024(−0.028 to 0.020)	*z*_1_, *z*_2_, *z*_5_, *z*_6_	
3	0.149±0.103(0.046 to 0.253)	−0.057±0.113(−0.170 to 0.055)	*z*_1_, *z*_2_, *z*_5_, *z*_6_	
4	0.117±0.058(0.059 to 0.175)	0.005±0.003(0.001 to 0.008)	*z*_1_, *z*_2_, *z*_5_, *z*_6_	
5	0.152±0.107(0.044 to 0.260)	−0.060±0.118(−0.179 to 0.057)	*z*_1_, *z*_2_, *z*_5_, *z*_6_	
	**Validation metrics**			
	RMSE (°C) Weirμ±σ (range)	RMSE (°C) Validationμ±σ (range)	MB (°C)μ±σ (range)	$E_{DUP}\;({}^{\circ}C)\;\mu \pm \sigma \;(\mathrm{range})$
Manufacturer calibration	2.171±0.350(1.821 to 2.519)	1.579±0.738(0.841 to 2.317)	−0.520±1.642(−2.162 to 1.121)	0.247±0.229(0.018 to 0.476)
1	0.454±0.113(0.340 to 0.567)	0.332±0.156(0.175 to 0.488)	0.078±0.293(−0.215 to 0.371)	0.222±0.166(0.056 to 0.388)
2	0.395±0.076(0.319 to 0.472)	0.292±0.141(0.151 to 0.433)	0.162±0.186(−0.024 to 0.349)	0.156±0.180(0.000 to 0.336)
3	0.488±0.077(0.410 to 0.566)	0.347±0.185(0.161 to 0.533)	0.218±0.247(−0.028 to 0.466)	0.251±0.232(0.019 to 0.483)
4	0.316±0.078(0.238 to 0.394)	0.250±0.105(0.144 to 0.355)	0.025±0.174(−0.148 to 0.200)	0.192±0.161(0.031 to 0.353)
5	0.433±0.108(0.324 to 0.541)	0.330±0.176(0.153 to 0.507)	0.187±0.244(−0.057 to 0.431)	0.315±0.233(0.081 to 0.548)

**Table 3:** Calibration metrics for the northern Chile data set: single-ended and double-ended calibration metrics for the RC151 borehole (calibration baths: *z*_1_, *z*_2_, *z*_5_, *z*_6_; validation baths: *z*_3_, *z*_4_), the DH009 borehole (calibration baths: *z*_1_, *z*_2_, *z*_3_, *z*_4_; validation baths: *z*_5_, *z*_6_), and the DDH2457 borehole (calibration baths: *z*_1_, *z*_2_, *z_4_*; validation baths: *z*_3_, *z*_4_, *z*_6_). The position of the reference baths along the cable, i.e., *z*_1_–*z*_6_, are shown in Figure 4.

RC151	Single-Ended Measurements		Double-Ended Measurements	
Metric	Calibration	Validation	Calibration	Validation
RMSE (°C)μ±σ (range, °C)	0.083±0.019 (0.064 to 0.102)	0.113±0.021 (0.092 to 0.134)	0.151±0.068 (0.083 to 0.219)	0.102±0.010 (0.092 to 0.113)
MB (°C)μ±σ (range, °C)	0.030±0.036 (−0.005 to 0.036)	0.090±0.024(−0.115 to−0.024)	−0.129±0.082(−0.047 to 0.211)	−0.028±0.003 (−0.031 to−0.024)
$E_{DUP}\;({}^{\circ}C)\;\mu \pm \sigma \;(\mathrm{range},\;{}^{\circ}C)$	0.512±0.460(0.052 to 0.973)		0.494±0.394(0.100 to 0.889)	
$E_{COMP}\;({}^{\circ}C)\;\mu \pm \sigma \;(\mathrm{range},\;{}^{\circ}C)$	0.063±0.047 (0.015 to 0.110)			
**DDH009**	**Single-Ended Measurements**		**Double-Ended Measurements**	
Metric	Calibration	Validation	Calibration	Validation
RMSE (°C)μ±σ (range, °C)	0.198±0.031(0.166 to 0.229)	0.290±0.098(0.192 to 0.388)	0.088±0.057(0.031 to 0.145)	0.104±0.020(0.084 to 0.125)
MB (°C)μ±σ (range, °C)	0.032±0.054(−0.021 to 0.086)	−0.032±0.000(−0.021 to 0.086)	0.005±0.016(−0.011 to 0.086)	0.096±0.021(0.075 to 0.118)
$E_{DUP}\;({}^{\circ}C)\;\mu \pm \sigma \;(\mathrm{range},\;{}^{\circ}C)$	0.445±0.322(0.122 to 0.767)		0.324±0.261(0.062 to 0.586)	
$E_{COMP}\;({}^{\circ}C)\;\mu \pm \sigma \;(\mathrm{range},\;{}^{\circ}C)$	0.094±0.101 (0 to 0.196)			
**DDH2457**	**Single-Ended Measurements**		**Double-Ended Measurements**	
Metric	Calibration	Validation	Calibration	Validation
RMSE (°C)μ±σ (range)	0.068±0.015(0.052 to 0.084)	0.106±0.024(0.081 to 0.130)	0.119±0.035 (0.084 to 0.153)	0.117±0.045 (0.072 to 0.163)
MB (°C)μ±σ (range)	−0.081±0.092 (−0.011 to 0.174)	0.055±0.053(0.005 to 0.105)	−0.047±0.055 (−0.103 to 0.008)	−0.015±0.077 (−0.092 to 0.062)
$E_{DUP}\;({}^{\circ}C)\;\mu \pm \sigma \;(\mathrm{range})$	0.297±0.295 (0.001 to 0.592)		0.260±0.234 (0.025 to 0.495)	
$E_{COMP}\;({}^{\circ}C)\;\mu \pm \sigma \;(\mathrm{range})$	0.073±0.052 (0.125 to 0.021)			

**Table 4:** Geothermal gradients estimated in the boreholes investigated in this study.

		Single-Ended Measurements (Algorithm 4)		Double-Ended Measurements	
Location	Borehole ID	Geothermal Gradient (°C km^−1^)	Temperature at Cable’s End (°C)	Geothermal Gradient (°C km^−1^)	Temperature at Cable’s End (°C)
Northern Chile	DDH2457	12.4	28.51	12.9	28.41
	RC151	10.4	26.09	10.5	26.05
	DDH009	9.7	28.71	10.2	28.97
Central Andes of Chile	DAND	37.9	38.71	-	-

**Table 5:** Metrics of the Central Chilean Andes dataset (calibration baths: *z*_1_, *z*_2_, *z*_3_, *z*_4_; validation baths: *z*_5_, *z*_6_). The location of the reference baths along the fiber, i.e., *z*_1_–*z*_6_, are shown in Figure 6.

Metric	Calibration	Validation
RMSE (°C) μ±σ (range)	0.115±0.051(0.064 to 0.166)	0.185±0.049 (0.136 to 0.235)
MB (°C) μ±σ (range)	$<10^{-4}$	0.143±0.045 (0.097 to 0.189)
$E_{DUP}\;({}^{\circ}C)\;\mu \pm \sigma$ (range)	0.223±0.149(0.073 to 0.372)	

## Data Availability

The raw and calibrated data are published as a dataset of this manuscript (http://dx.doi.org/10.17632/cytbff9vgt.2, accessed on 24 April 2022).

## Appendix A

Table A1 and Table A2 present the parameters used to estimate the thermal profile along the fiber-optic cable using the different algorithms in the laboratory deployment and in the boreholes measured.

**Table A1:** Calibration parameters obtained in the laboratory deployment. Reported values correspond to the mean ± standard deviation.

Algorithm	*C*_1_ (-) (Range)	*C*_2_ (-) (Range)	*γ*_1_ (K) (Range)	*γ*_2_ (K) (Range)	Δ*α*_1_ × 10^5^ (m^−1^) (Range)	Δ*α*_2_ × 10^5^ (m^−1^) (Range)
1	1.083 ± 0.002 (1.081 to 1.086)	–	483.2 ± 0.4 (482.8 to 483.6)	–	8.007 ± 0.048 (7.959 to 8.055)	–
2	1.083 ± 0.002 (1.081 to 1.086)	1.075 ± 0.003 (1.072 to 1.078)	483.2 ± 0.4 (482.8 to 483.6)	479.6 ± 0.7 (478.9 to 480.2)	8.007 ± 0.048 (7.959 to 8.055)	8.269 ± 0.066 (8.203 to 8.335)
3	1.083 ± 0.002 (1.081 to 1.086)	–	483.0 ± 0.4 (482.6 to 483.4)	–	8.098 ± 0.047 (8.053 to 8.143)	8.027 ± 0.046 (7.980 to 8.073)
4	1.083 ± 0.002 (1.081 to 1.086)	–	483.2 ± 0.4 (482.8 to 483.6)	480.2 ± 0.5 (479.6 to 480.7)	8.007 ± 0.048 (7.959 to 8.055)	8.616 ± 0.153 (8.511 to 8.772)
5	1.083 ± 0.002 (1.081 to 1.086)	1.084 ± 0.002 (1.081 to 1.086)	483.2 ± 0.4 (482.8 to 483.6)	–	8.007 ± 0.048 (7.959 to 8.147)	8.102 ± 0.048 (8.057 to 8.147)

**Table A2:** Calibration parameters obtained in the borehole deployments for Algorithm 4. Reported values correspond to the mean ± standard deviation.

Borehole	*C* (-) (Range)	*γ*_1_ (K) (Range)	*γ*_2_ (K) (Range)	Δ*α*_1_ × 10^5^ (m^−1^) (Range)	Δ*α*_2_ × 10^5^ (m^−1^) (Range)
DDH2457	1.542 ± 0.006 (1.536 to 1.548)	484.2 ± 0.4 (483.9 to 484.6)	484.0 ± 0.4 (483.6 to 484.5)	5.847 ± 0.059 (5.788 to 5.906)	5.896 ± 0.057 (5.838 to 5.953)
RC151	1.623 ± 0.018 (1.605 to 1.642)	496.3 ± 2.5 (493.8 to 498.9)	496.6 ± 2.4 (494.3 to 499.0)	5.639 ± 0.079 (5.560 to 5.718)	5.566 ± 0183 (5.383 to 5.750)
DDH009	1.576 ± 0.021 (1.555 to 1.597)	489.2 ± 1.5 (487.7 to 490.6)	489.0 ± 1.5 (487.5 to 490.6)	5.612 ± 0.159 (5.453 to 5.771)	5.642 ± 0.161 (5.481 to 5.803)
DAND	1.061 ± 0.007 (1.054 to 1.068)	474.0 ± 2.2 (471.8 to 476.2)	473.7 ± 2.2 (471.5 to 475.9)	8.414 ± 0.033 (8.381 to 8.447)	8.506 ± 0.037 (8.469 to 8.544)
